# Effects of (−)-Gallocatechin-3-Gallate on Tetrodotoxin-Resistant Voltage-Gated Sodium Channels in Rat Dorsal Root Ganglion Neurons

**DOI:** 10.3390/ijms14059779

**Published:** 2013-05-07

**Authors:** Yan Zhang, Yan-Yan Jia, Jin-Lei Guo, Pei-Qing Liu, Jian-Min Jiang

**Affiliations:** Laboratory of Pharmacology and Toxicology, School of Pharmaceutical Sciences, Sun Yat-Sen University, Guangzhou 510006, China; E-Mails: zhyan39@yahoo.com (Y.Z.); jiayanyan1018@126.com (Y.-Y.J.); guojinl@mail2.sysu.edu.cn (J.-L.G.); liupq@mail.sysu.edu.cn (P.-Q.L.)

**Keywords:** catechins, (−)-gallocatechin-3-gallate, Na^+^ channel, dorsal root ganglion, tetrodotoxin-resistant

## Abstract

The (−)-gallocatechin-3-gallate (GCG) concentration in some tea beverages can account for as much as 50% of the total catechins. It has been shown that catechins have analgesic properties. Voltage-gated sodium channels (Nav) mediate neuronal action potentials. Tetrodotoxin inhibits all Nav isoforms, but Nav1.8 and Nav1.9 are relatively tetrodotoxin-resistant compared to other isoforms and functionally linked to nociception. In this study, the effects of GCG on tetrodotoxin-resistant Na^+^ currents were investigated in rat primary cultures of dorsal root ganglion neurons via the whole-cell patch-clamp technique. We found that 1 μM GCG reduced the amplitudes of peak current density of tetrodotoxin-resistant Na^+^ currents significantly. Furthermore, the inhibition was accompanied by a depolarizing shift of the activation voltage and a hyperpolarizing shift of steady-state inactivation voltage. The percentage block of GCG (1 μM) on tetrodotoxin-resistant Na^+^ current was 45.1% ± 1.1% in 10 min. In addition, GCG did not produce frequency-dependent block of tetrodotoxin-resistant Na^+^ currents at stimulation frequencies of 1 Hz, 2 Hz and 5 Hz. On the basis of these findings, we propose that GCG may be a potential analgesic agent.

## 1. Introduction

**Figure 1:**
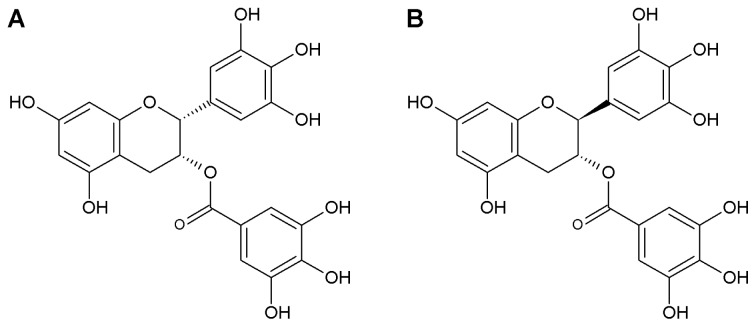
Chemical structures of (−)-Epigallocatechin-3-gallate (EGCG) (**A**) and (−)-Gallocatechin-3-gallate (GCG) (**B**).

Green tea has many pharmacological actions mainly attributed to catechins. Studies have demonstrated an antinociceptive effect of green tea extract on inflammatory and neuropathic pain [1,2]. (−)-Epigallocatechin-3-gallate (EGCG) is one of the major catechins of green tea leaves and it has been shown to exhibit analgesic efficacy. It has been suggested that EGCG produces an antiallodynic effect against neuropathic pain by blocking the increase in nitric oxide synthase (NOS) expression in the spinal cord and thus inhibiting the pronociceptive effects of nitric oxide (NO) [3]. Moreover, EGCG dramatically improved pain behaviors in a rat chronic constriction injury model of neuropathic pain [4]. (−)-Gallocatechin-3-gallate (GCG) is produced following the epimerization of EGCG during the heating procedure (Figure 1). Therefore, the stability of GCG is much better than that of EGCG. Studies have shown that GCG has the most prolonged hypotensive effect in rabbits among the catechins [5], and the inhibitory effect of GCG on cholesterol absorption was more effective than that of EGCG [6]. However, GCG has received relatively little attention because its content in green tea is much less than EGCG. Recently, instead of EGCG, GCG was found to be the predominant catechin in cocoa tea, which is a naturally decaffeinated tea plant growing in southern China [7]. It is therefore necessary to investigate the analgesic efficacy of GCG.

Voltage-gated sodium channels (Nav), which produce the inward membrane current necessary to produce regenerative action potentials in neurons and muscle cells, have emerged as important targets in the study of the molecular pathophysiology of pain and in the search for new pain therapies [8]. Nociceptive neurons within dorsal root ganglia (DRG) express multiple Na^+^ channel subtypes that can be separated into two groups based on their susceptibility to tetrodotoxin (TTX) [9]. Small-diameter (<25 μm) rat DRG neurons express a combination of fast tetrodotoxin-sensitive (TTX-S) and slow tetrodotoxin-resistant (TTX-R) Na^+^ currents while large-diameter (>30 μm) neurons predominately express fast tetrodotoxin-sensitive Na^+^ currents [10]. Tetrodotoxin-resistant Na^+^ currents show distinctive biophysical properties, such as persistent and slowly inactivating currents. The persistent current has been attributed to Nav1.9, and the slowly inactivating current to Nav1.8. By transmitting the majority of nociceptive signals to the spinal cord, small to medium-sized DRG neurons play an important role in pain sensory detection.

Previous studies have found that EGCG potently inhibited tetrodotoxin-sensitive and tetrodotoxin-resistant Na^+^ currents in rat DRG neurons [10], and Na^+^ currents in rat hippocampal neurons in a concentration-dependent manner [11]. We therefore sought to investigate whether GCG could suppress Na^+^ currents passing through nociceptive-related tetrodotoxin-resistant Na^+^ channels in rat DRG neurons, and if so, whether the inhibition was frequency-dependent. The results suggest that GCG may reduce pain peripherally through inhibition of tetrodotoxin-resistant Na^+^ currents in nociceptive sensory neurons.

## 2. Results and Discussion

### 2.1. GCG Inhibits Tetrodotoxin-Resistant Na^+^ Currents in Dorsal Root Ganglion Neurons

**Figure 2:**
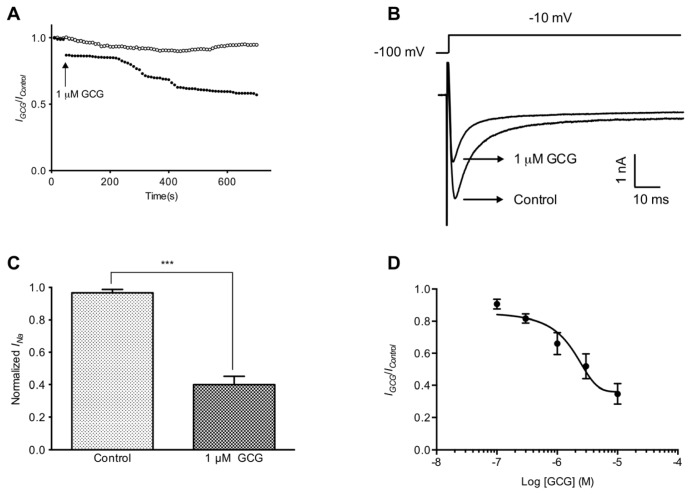
GCG-induced inhibitory effect on tetrodotoxin-resistant Na^+^ currents in rat dorsal root ganglia (DRG) neurons. (**A**) Example time courses of tetrodotoxin-resistant Na^+^ current in the absence (unfilled circles) and presence of 1 μM GCG (filled circles); (**B**) Representative tetrodotoxin-resistant Na^+^ currents in rat DRG neurons; (**C**) The blockade of tetrodotoxin-resistant Na^+^ currents after treatment with 1 μM GCG for 5 min (*n* = 6); (**D**) Concentration-response curve of tetrodotoxin-resistant Na^+^ currents inhibition by GCG (*n* = 4).

We have focused on testing the effect of GCG on tetrodotoxin-resistant Na^+^ channels, which were isolated from tetrodotoxin-sensitive Na^+^ currents by the application of 100 nM tetrodotoxin. A low (60 mM) Na^+^ bath solution was used to reduce the current amplitude in order to minimize voltage-clamp errors. We used a tonic block protocol to assess the degree of tetrodotoxin-resistant Na^+^ current inhibition after long time exposure (30 min) to 1 μM GCG (Figure 2A). Tetrodotoxin-resistant Na^+^ currents were activated by stepping from a holding potential of −100 to −10 mV and recorded at 10 s intervals (Figure 2B). GCG significantly reduced the amplitude of the tetrodotoxin-resistant Na^+^ currents in a time-dependent manner. In the presence of 100 nM TTX, the residual sodium currents (regarded as tetrodotoxin-resistant Na^+^ currents) were initially slightly reduced by 1 μM GCG. The blockage of tetrodotoxin-resistant Na^+^ currents by 1 μM GCG developed slowly and reached its maximum about 400 s later. Finally, GCG appeared to block half the tetrodotoxin-resistant Na^+^ currents in 10 min. The decrease, however, was statistically significant with 45.1% ± 1.1% (*p* < 0.005, *n* = 6) (Figure 2C).

The concentration-response relationship is shown in Figure 2D and the IC_50_ value was calculated to be 1.4 μM for tetrodotoxin-resistant Na^+^ currents (*n* = 4).

### 2.2. Effects of GCG on Tetrodotoxin-Resistant Na^+^ Current Activation

**Figure 3:**
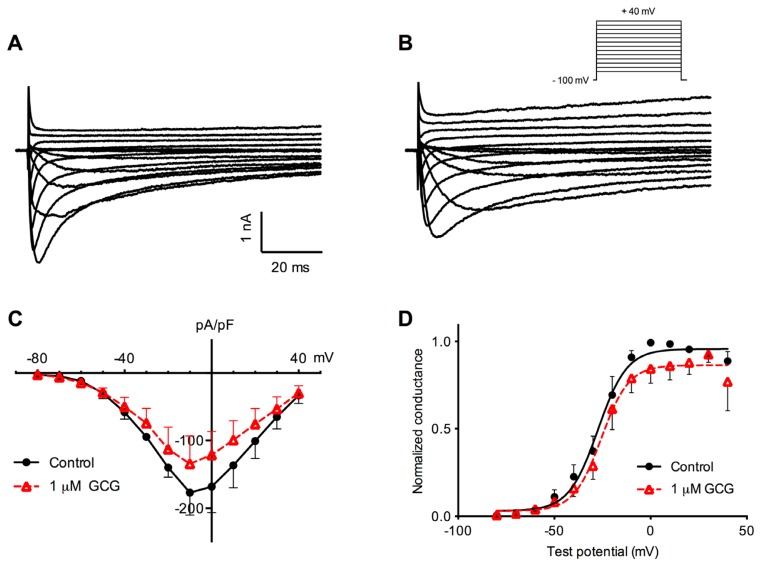
Effects of GCG on the activation of tetrodotoxin-resistant Na^+^ currents. (**A**,**B**) Original recordings of tetrodotoxin-resistant Na^+^ currents under control condition (**A**) and after treatment with 1 μM GCG (**B**); (**C**) Averaged current-voltage (*I*–*V*) curves of tetrodotoxin-resistant Na^+^ currents before (filled circle) and after (unfilled triangle) 1 μM GCG application; (**D**) Conductance-voltage (*G*-*V*) relationships derived from *I–V* data (*n* = 6).

We next examined if GCG affected tetrodotoxin-resistant Na^+^ channel gating. After establishing the whole-cell configuration, neurons were voltage-clamped at −100 mV. Tetrodotoxin-resistant Na^+^ currents were evoked by depolarizing step pulses from −80 to +40 mV in steps of 10 mV. Examples of tetrodotoxin-resistant Na^+^ currents recorded in the absence and presence of 1 μM GCG for 5 min are shown in Figure 3A,B. The current-voltage (*I–V*) relationship curve for tetrodotoxin-resistant Na^+^ currents averaged from six cells is shown in Figure 3C. The current activation threshold was approximately −60 mV and peak inward current occurred at approximately −10 mV. GCG, at a 1 μM concentration, blocked peak current density from −177.0 ± 32.9 to −134.6 ± 41.2 pA/pF (*p* < 0.05, *n* = 6).

The activation of tetrodotoxin-resistant Na^+^ channels was fitted with a Boltzmann function, as shown in Figure 3D. GCG had some influence on the voltage-dependence of activation of the tetrodotoxin-resistant Na^+^ current. The activation of tetrodotoxin-resistant Na^+^ channels was fitted with a Boltzmann function, as shown in Figure 3D. GCG had some influence on the voltage-dependence of activation of the tetrodotoxin-resistant Na^+^ current. *V*_1/2_ and *k* were calculated to be −27.4 ± 1.3 mV and 7.5 ± 1.2 mV in control (*n* = 6), respectively. GCG (1 μM) treatment for 5 min produced a slight but significant depolarizing shift of *V*_1/2_ to 25.6 ± 1.3 mV (*p* < 0.05). However, the slope factor *k* was not change significantly by GCG treatment.

### 2.3. Effects of GCG on Steady-State Inactivation Tetrodotoxin-Resistant Na^+^ Currents

**Figure 4:**
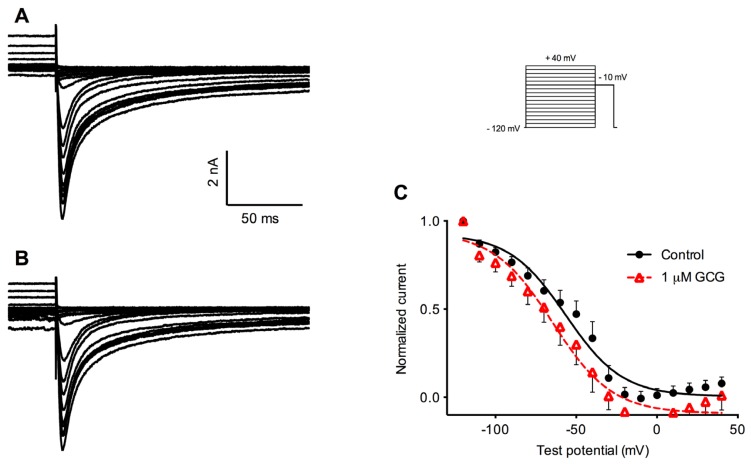
Effects of GCG on the steady-state inactivation of tetrodotoxin-resistant Na^+^ currents. (**A**,**B**) Original recordings of tetrodotoxin-resistant Na^+^ currents elicited by depolarizing to −10 mV after a 1.5 s conditioning prepulse ranging from −120 mV to +40 mV under control condition (**A**) and after treatment with 1 μM GCG for 5 min (**B**); (**C**) Steady-state inactivation curves in control (filled circles) and in the presence (unfilled triangles) of 1 μM GCG (*n* = 5).

Next, we examined whether GCG had any effect on voltage-dependent inactivation of tetrodotoxin-resistant Na^+^ currents. After a 1.5 s conditioning prepulse ranging from −120 to +40 mV, the steady-state inactivation was assessed by stepping the voltage to −10 mV. Figure 4 illustrates the steady-state inactivation voltage protocol and examples of tetrodotoxin-resistant Na^+^ currents recorded using this protocol before (Figure 4A) and after the application of 1 μM GCG for 5 min (Figure 4B). The steady-state inactivation parameter was fitted to a Boltzmann function. The *V*_1/2_ for tetrodotoxin-resistant Na^+^ current was calculated to be −56.5 ± 3.9 mV in control (*n* = 5). GCG, at 1 μM, shifted *V*_1/2_ to −64.6 ± 4.4 mV, which is more negative than for control (*p* < 0.01). As with the activation curves, there was no statistically significant change in the slope factor *k* (Figure 4C).

### 2.4. Frequency-Dependent Block of Tetrodotoxin-Resistant Na^+^ Currents

**Figure 5:**
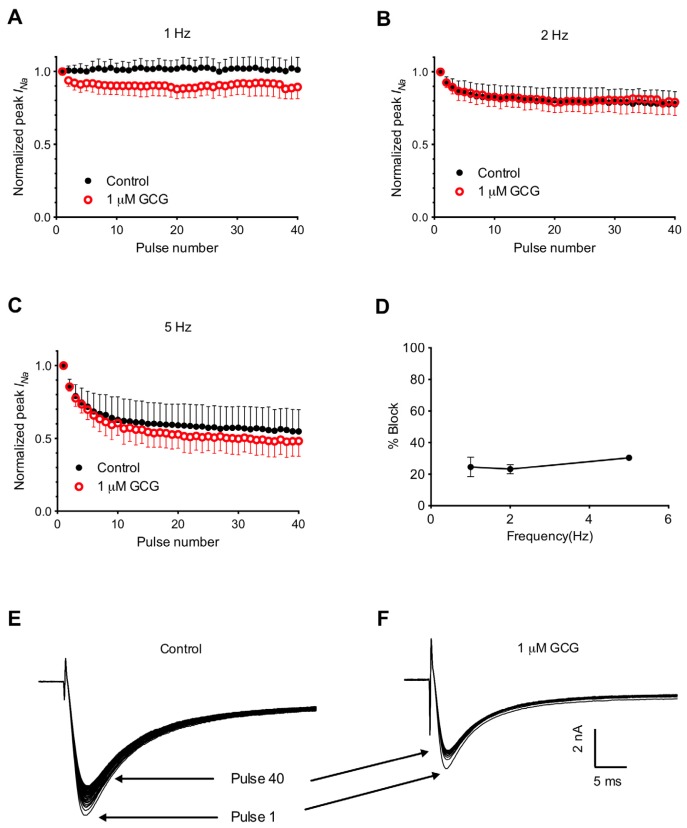
The frequency-dependen blockage effect on tetrodotoxin-resistant Na^+^ currents. Whole-cell currents were evoked by 40-step depolarization commands (holding potential −120 mV; test potential −10 mV; pulse duration 40 ms). (**A**–**C**) The ratios of the current amplitude at the testing pulse *versus* the 1st pulse are shown at 1 Hz (**A**), 2 Hz (**B**) and 5 Hz (**C**) in the absence (filled circle) and presence (unfilled circle) of 1 μM GCG; (**D**) The percentage block of 1 μM GCG on tetrodotoxin-resistant Na^+^ currents at different frequencies (*n* = 5 for each frequency). No frequency-dependent effect was observed (ANOVA, *p* > 0.05). (**E**,**F**) Representative recordings at 2 Hz in the absence (**E**) or presence (**F**) of 1 μM GCG. Arrows mark traces for pulse 1 and pulse 40.

To further characterize the frequency-dependent blockade of GCG on tetrodotoxin-resistant Na^+^ currents, we applied pulse trains of various frequencies (1, 2 and 5 Hz), with each train containing 40 pulses (Figure 5A–C). At 1 Hz, 2 Hz and 5 Hz, the current amplitude ratios in the absence and in the presence of 1 μM GCG are almost parallel, suggesting that there is little or no frequency-dependent blockade of GCG on tetrodotoxin-resistant Na^+^ currents. The percentage block of 1 μM GCG on tetrodotoxin-resistant Na^+^ currents was calculated using the following formula: Percentage Block = 100 – 100(*I*_GCG_/*I*_control_), where *I*_GCG_ is the current amplitude measured at the 40th pulse in the presence of 1 μM GCG, and *I*_control_ is the current amplitude measured at the 40th pulse under control conditions in the same cell. Figure 5D shows the averaged block (*n* = 5) at three different pulse frequencies. ANOVA analysis indicated that there was no frequency-dependent block of GCG on tetrodotoxin-resistant Na^+^ currents.

### 2.5. Discussion

In the present study we show that GCG potently inhibited tetrodotoxin-resistant Na^+^ currents in DRG neurons at a concentration of 1 μM. GCG slightly shifted the activation curves of tetrodotoxin-resistant Na^+^ currents in the depolarized direction, but more significantly shifted the steady-state inactivation curves of tetrodotoxin-resistant Na^+^ currents in the hyperpolarized direction. These data suggest that GCG reduced the availability for activation and enhanced the inactivation of tetrodotoxin-resistant Na^+^ channels. The effects of GCG on tetrodotoxin-resistant Na^+^ channels are not frequency-dependent, which could be explained by its fast dissociation from blocked channels during repolarization due to its hydrophilic nature. This, however, should not influence the analgesic effects of GCG. As C-fibers have relatively low action potential firing frequencies, the frequency dependence of a Na^+^ channel blocker is expected to be of limited importance for analgesia [12,13]. Overall, These results suggest that GCG is able to block tetrodotoxin-resistant Na^+^ currents and therefore, potentially interfere with pain transduction pathways.

We have demonstrated that GCG is a low-affinity inhibitor of tetrodotoxin-resistant Na^+^ channels with electrophysiological properties that are similar to those of its epimer EGCG [14,15]. The inhibitory effect of GCG exhibited slow kinetics and approached steady-state conditions only after about 5 min. As a result, it was difficult to determine an equilibrium dose-response curve for GCG blockade of tetrodotoxin-resistant Na^+^ currents in rat small DRG neurons. We take into account the dose-dependence of EGCG on electrical activity in DRG neurons and other tissues that have previously been reported. It has been shown that voltage-dependent Na^+^ currents were much more sensitive to EGCG in DRG cells, being inhibited to 43% ± 6% of control by 1 μM EGCG [10]. In contrast, another study found that voltage-dependent Na^+^ channels in hippocampal neurons were only weakly inhibited by EGCG with an IC_50_ value of approximately 200 μM [11]. Likewise, the human ether-a-go-go-related gene (HERG) cardiac K^+^ channel was inhibited by EGCG with an IC_50_ value of 6 μM while another study found that EGCG was only a weak inhibitor of HERG [14,15]. The discrepancy of the EGCG effects possibly stems from the different tissues studied. In our result, at a 1 μM concentration, GCG decrease tetrodotoxin-resistant Na^+^ currents to 45.1% ± 1.1% of control, in line with a previous study which reported that applying EGCG (1 μM) for 10 min inhibited tetrodotoxin-resistant Na^+^ currents in DRG neurons to 43% ± 6% of control. According to previous reports, GCG had a more positive inotropic effect [5], and displayed equal cardioprotective effects by administration of the lower concentration than that of EGCG [16]. Moreover, GCG on up-regulating of the LDL receptor is significantly greater than that of EGCG [17], consistent with a report that GCG-rich tea catechins are more effective in inhibiting cholesterol absorption than EGCG-rich tea catechins [6]. However, no significant higher potency was found in voltage-clamp experiments.

It has been reported that EGCG had little or no effect on the activation or steady-state inactivation voltage of tetrodotoxin-resistant Na^+^ current in DRG neurons, but that slope factors were significantly shifted by EGCG [10]. In another study, EGCG caused a hyperpolarizing shift of both activation and steady-state inactivation voltages of tetrodotoxin-sensitive Na^+^ current in hippocampal neurons [11]. In the present study, we observed that GCG shifted the activation voltage towards more positive potentials, but more significant negative shift of steady-state inactivation was found. In addition, there were no significant effects on slope values. Voltage-dependent block by EGCG results in a hyperpolarizing shift in steady-state inactivation, thus enhancing channel block at normal as opposed to hyperpolarized potentials. GCG apparently inhibits tetrodotoxin-resistant Na^+^ channels by a similar mechanism involving enhanced inactivation.

Catechins have very low bioavailability. Following the oral ingestion of 350 mL of beverage containing 581 mg green tea catechins, optimal LC-ESIMS analysis revealed that the concentrations of EGCG and GCG in human plasma were 261.2 ± 79.1 and 14.6 ± 9.6 ng h/mL (concentration *versus* time profiles) whereas their concentrations in the green tea beverage ingested were 0.31 and 0.36 mg/mL, respectively [18]. EGCG has been reported as producing the highest catechin concentration in human plasma after oral ingestion of a commercial green tea beverage [18,19]. Polyphenon E (a decaffeinated and defined green tea catechin mixture), up to a dose that contains 800 mg EGCG, is well-tolerated when taken under fasting conditions [20]. It was also found that concentrations of EGCG and GCG declined rapidly after intravenous dosing and much smaller intravenous dose was required to achieve a maximum concentration comparable to that of an oral dose. Maximum concentration values of EGCG and GCG were 17.0 and 20.4 μg/mL plasma at 36 and 40 min respectively after the oral dosing in rat [21]. However, there is as yet no information about the intrathecal concentration of GCG required to produce analgesia. Additionally, studies in humans are also needed to better understand the dose-response relationships in the plasma levels and in urinary excretion of GCG after ingestion of tea. Similar to EGCG, GCG is highly hydrophilic due to its OH-groups, and hence the unbound plasma concentration may be assumed to approximate. GCG-rich tea catechins have been manufactured, in which GCG concentration was as high as 50% of the total catechins from the process of heat epimerization. It is suggested that voltage-dependent ion channels could, at least in some tissues, be the pharmacological targets for the effects of GCG.

## 3. Experimental Section

### 3.1. Dissociation and Culture of Dorsal Root Ganglion Neurons

All experiments were conducted in accordance with the guidelines and the approval of the Ethics Committee for Animal Use of Sun Yat-sen University. Newborn (2–6 days old) Sprague-Dawley rats were decapitated, and DRGs were rapidly removed from spinal cord and put in ice cold Ca^2+^/Mg^2+^-free phosphate-buffered saline (PBS). The isolated DRGs were digested at 36 °C first in Dulbecco’s Modified Eagle Medium (DMEM) containing 0.1% collagenase (Type II, Gibco, Invitrogen Corporation, Carlsbad, CA, USA) for 15 min and then transferred to PBS containing 0.25% trypsin (TypeXI, Sigma, St. Louis, MO, USA) and incubated further for 10 min. Single DRG cell suspensions were obtained by gentle trituration and the final dissociated cells were plated onto poly-d-lysine-coated coverslips and were cultured in DMEM medium supplemented with 10% fetal bovine serum, 10,000 units penicillin and 10 mg/mL streptomycin (all from Gibco) and 50 ng/mL nerve growth factor (NGF). Electrophysiological experiments were performed approximately 2–7 h after dissociation.

### 3.2. Electrophysiology

Voltage-clamp recordings were made in the whole-cell mode using an Axopatch-200B amplifier (Axon Instruments, Sunnyvale, CA, USA), and current was digitized using a Digidata 1440A interface (Axon Instruments, Sunnyvale, CA, USA). Fire-polished patch pipettes were pulled from borosilicate glass capillaries (Warner Instruments, Hamden, CT, USA) by using a Sutter P-97 puller (Sutter Instruments, Novato, CA, USA) and had a resistance of 2–4 MΩ when filled with the following internal pipette solution (in mM): 120 CsF, 10 NaCl, 10 HEPES, 11 EGTA, 10 tetraethylammonium-Cl, 1 CaCl_2_, 1 MgCl_2_, adjusted to pH 7.3 (with CsOH) and 315 mOsm/kg H_2_O. Voltage protocols were generated by using pClamp10.1 (Axon Instruments) software. Data were digitized at 10 or 20 kHz and analyzed using Clampfit 10.1 (Axon Instruments) and Prism 5.0 softwares (GraphPad). Pipette potentials were zeroed before seal formation. Liquid junction potentials were not corrected. The capacitance of DRG neurons in our study was less than 30 pF. Series resistance was less than 10 MΩ and we routinely compensated up to 70% of the series resistance error. Cells with a series resistance greater than 10 MΩ were discarded. Linear leakage currents were digitally subtracted online by the P/4 protocol (except in inactivation and frequency-dependent experiments). The low Na^+^ external solution was used for DRG Na^+^ current recordings, contained (in mM): 60 NaCl, 80 KCl, 2 CaCl_2_, 2 MgCl_2_, 10 HEPES, and 10 glucose (pH = 7.4 adjusted with NaOH) and 310 mOsm/kg H_2_O. CdCl_2_ (200 μM) was used to block Ca^2+^ currents. All chemicals were from Sigma, unless otherwise stated. All experiments were conducted at room temperature (22 ± 2 °C).

Tetrodotoxin-resistant Na^+^ current was present primarily in small-diameter neurons (<20 μm). To isolate tetrodotoxin-resistant Na^+^ current, 100 nM TTX (Sigma) was included in the bath solution in all experiments. GCG (Sigma, St. Louis, MO, USA) was dissolved in dimethylsulfoxide (DMSO) at a stock concentration of 10 mM. Stock solutions were stored frozen at −20 °C in small aliquots and diluted in the bath solution to the desired concentrations immediately before use. A recording chamber with volume of ~0.5 mL was continuously perfused with the bath solution by gravity at a rate of ~1 mL/min.

### 3.3. Statistical Analysis

Pooled data are presented as means ± S.E.M. Statistical comparisons were made using two-way analysis of variance (ANOVA) and two-tailed *t* test with Bonferroni correction. *p* < 0.05 was considered statistically significant.

## 4. Conclusions

The present study is the first detailed investigation on the effects of GCG on pain related ion channels. We showed that GCG inhibited tetrodotoxin-resistant Na^+^ currents in rat DRG neurons potently, and at a concentration that can be reached physiologically. The critical role of tetrodotoxin-resistant Na^+^ channels in peripheral pain mechanisms suggests that its inhibition could contribute to the antinociceptive and thus GCG has a potential for use as an analgesic agent.
